# Commentary: Prognostic nutritional index and inflammatory indices as predictors of perioperative infection in patients with Cushing syndrome: a retrospective single-center study

**DOI:** 10.3389/fendo.2026.1883925

**Published:** 2026-07-28

**Authors:** Liqing Wu, Ping Yan

**Affiliations:** Hangzhou Fuyang Hospital of TCM Orthopedics and Traumatology, Hangzhou, China

**Keywords:** cushing syndrome, perioperative infection, prognostic nutritional index, risk prediction, ROC-derived cut-offs

We read with interest the study by Chasan et al. examining the prognostic value of cortisol-related indices, blood cell-derived inflammatory indices, and the prognostic nutritional index for perioperative infection in patients with endogenous Cushing syndrome (1). Perioperative infection in a high-cortisol and immunologically vulnerable surgical population is clinically important and remains insufficiently studied. By focusing on this problem, the authors provide useful hypothesis-generating evidence for perioperative risk stratification. In particular, the observed signals for the 1-mg dexamethasone suppression test (1-mg DST) and the prognostic nutritional index (PNI) are clinically attractive because they are readily available, interpretable, and potentially actionable in routine perioperative assessment.

The methodological points raised below should therefore not be interpreted as criticism of this study in isolation. Univariable screening before multivariable modelling, in-sample receiver operating characteristic (ROC)-derived cut-offs, and dichotomization of continuous markers are common across the clinical prediction and perioperative-risk literature, especially in single-center retrospective studies with limited events. The study by Chasan et al. is representative of current practice rather than an outlier. Our intention is to use this clinically relevant work as an opportunity to highlight field-wide methodological habits that may affect the interpretation, reproducibility, and transportability of risk prediction findings.

First, the variable-selection strategy used for the multivariable logistic regression model deserves cautious interpretation. The authors included 113 patients, of whom 35 developed perioperative infection, and evaluated multiple clinical, hormonal, inflammatory and nutritional variables. Multivariable models were constructed after univariable screening, and the “best-performing model” was selected as the final model. This strategy may be unstable in a relatively small cohort with a limited number of outcome events. This approach is frequently used in retrospective clinical studies, but it may be unstable when the number of events is limited relative to the number of candidate predictors (2). This concern is particularly relevant because several candidate predictors were biologically and statistically correlated. For example, 1-mg dexamethasone suppression test, urinary free cortisol, late-night salivary cortisol and low-dose dexamethasone suppression test all reflect cortisol burden, while PNI, NLR, SII and PIV share overlapping hematological components, particularly lymphocyte counts. Under such conditions, stepwise selection may select variables partly on the basis of random sample variation rather than robust independent predictive value. With only 35 infection events and a broad set of candidate predictors, the effective events-per-variable ratio was low, approximately two to three events per candidate predictor if the full candidate set is considered. A prespecified model based on clinical rationale, penalized regression such as LASSO, or bootstrap-based internal validation would represent better practice for future model development (3). However, these methods are not mechanical fixes for sparse data; in such a small cohort, even penalized or resampling-based approaches may remain unstable. They should therefore be framed as better practice for future validation and model development, rather than as procedures that would necessarily have changed the conclusions of the present study.

Second, the use of ROC-derived cut-off values followed by logistic regression in the same dataset may introduce optimism bias. The authors identified optimal thresholds using the Youden index, including 1-mg DST ≥17.2 µg/dL, PNI ≤51.4, NLR >3.43, SII >1141 and PIV >745.5, and then used these dichotomized variables in regression analyses. This approach can overestimate both discrimination and effect estimates because the cut-off values are derived and tested in the same sample. The large odds ratios reported for PNI ≤51.4 and 1-mg DST ≥17.2 µg/dL may therefore be influenced by data-driven threshold selection, sparse events, and sampling variability. Without reanalysis or external validation, however, it would be too strong to conclude that these estimates reflect instability rather than true effects. A more balanced interpretation is that the direction and clinical plausibility of the associations are informative, whereas the exact cut-offs and effect-size magnitudes should be considered preliminary. In addition, dichotomizing continuous predictors reduces statistical information, assumes an abrupt risk change at an arbitrary threshold and may impair transportability to other cohorts (4). For future work, modelling PNI, 1-mg DST, and related biomarkers as continuous variables, preferably with flexible approaches such as restricted cubic splines to allow for non-linearity, would preserve statistical information and avoid assuming an abrupt risk change at a single threshold (4). Candidate thresholds, if clinically useful, should then be validated using bootstrap resampling, cross-validation, or preferably external cohorts. From a practical standpoint, the reported cut-offs should not be treated as definitive decision rules. They can nevertheless serve as reasonable starting points for perioperative risk awareness, prompting closer infection surveillance, nutritional assessment, optimization of hypercortisolism, and individualized perioperative management while awaiting external validation.

Third, the small ectopic Cushing syndrome subgroup should be interpreted with caution. The final cohort included only five patients with ectopic Cushing syndrome, compared with 71 patients with pituitary Cushing syndrome and 37 with adrenal Cushing syndrome. Ectopic Cushing syndrome is typically characterized by more severe hypercortisolism and a higher risk of infectious complications. In such a small subgroup, a change in only one patient can substantially alter subgroup proportions, P values and model estimates. Therefore, the observed differences in infection frequency, cortisol burden, inflammatory indices and hypokalaemia among etiological subgroups may be statistically unstable. Sensitivity analyses excluding patients with ectopic Cushing syndrome, or combining etiological subtype adjustment with caution, would help clarify whether the main signals for PNI and 1-mg DST are independent of this small but clinically severe subgroup.

In summary, the study by Chasan et al. addresses an important and under-investigated perioperative problem and identifies clinically plausible signals that should not be dismissed. Rather than viewing the methodological limitations as isolated errors, they may be better understood as examples of broader practices that remain common in retrospective perioperative risk prediction studies. The findings for PNI and 1-mg DST are best interpreted as hypothesis-generating signals that may inform risk awareness and future model development, not as fully validated prediction rules. Additional analyses examining continuous predictor effects, internal optimism, and the influence of the ectopic subgroup, followed by multicenter external validation, would further strengthen the clinical interpretability and generalizability of this valuable work.
